# Design and methods of a multicenter randomized clinical trial of effects of diabetes-educated psychologist on glucose management and diabetes distress

**DOI:** 10.3389/fcdhc.2025.1549234

**Published:** 2025-04-16

**Authors:** Johanna Zeijlemaker, Therese Anderbro, Sofia Sterner Isaksson, Marcus Lind

**Affiliations:** ^1^ Department of Molecular and Clinical Medicine, Institute of Medicine, Sahlgrenska Academy, University of Gothenburg, Gothenburg, Sweden; ^2^ Department of Psychology, Stockholm University, Stockholm, Sweden; ^3^ Department of Medicine, NU Hospital Group, Uddevalla, Sweden; ^4^ Department of Medicine, Sahlgrenska University Hospital, Gothenburg, Sweden

**Keywords:** type 1 diabetes (T1D), diabetes distress, hemoglobin A1c (HbA1c), diabetes-educated psychologist, cognitive behavioral therapy (CBT)

## Abstract

**Introduction:**

Many people with type 1 diabetes struggle to manage their glucose levels and experience stress related to the behavioral demands of the disease. The aim of this study is to investigate whether treatment with a diabetes-educated psychologist can improve glucose levels and decrease diabetes distress.

**Materials and methods:**

Individuals with HbA1c >62 mmol/mol (7.8%) were randomized to either psychological treatment or control group. The study duration for each participant was 52 weeks. Patients who received treatment met with a diabetes-educated psychologist a minimum of seven times. In total 6 outpatient diabetes units and 10 psychologists participated. Cognitive behavioral therapy was primarily the treatment of choice. Both groups met with a diabetes nurse and/or physician at the start of the study and at 3, 6, and 12 months. HbA1c, blood pressure, and weight were measured at scheduled visits. Diabetes distress, quality of life, hypoglycemia confidence, and treatment satisfaction were evaluated using questionnaires. The primary endpoint is the difference in HbA1c from baseline to week 52. Secondary endpoints are changes in diabetes distress and quality of life from baseline to week 52, as well as treatment satisfaction at 52 weeks.

**Discussion:**

This study seeks to improve knowledge about how to support patients who struggle to manage their diabetes. If the results of this study show that psychological treatment has an effect on HbA1c or on diabetes distress, it could indicate that psychologists should become more involved in diabetes care teams. Clinical trial registration: ClinicalTrials.gov ID NCT03753997

## 1 Introduction

Type 1 diabetes with suboptimal glucose regulation is connected to a number of serious complications such as cardiovascular disease, retinopathy, neuropathy and nephropathy (1). Research has shown that good glycemic management can significantly decrease risk for complications and mortality (2). There are various strategies to achieve optimal HbA1c, including continuous glucose monitors (CGMs), multiple daily insulin injections (MDI), and insulin pumps. However, many patients still struggle to meet target blood glucose levels.

Recommendations for people with diabetes include adopting a number of self-care behaviors to manage the disease, which many people find challenging (3, 4). This includes frequent monitoring of blood glucose levels as well as following extensive recommendations about diet and exercise (5). Since these behaviors can be demanding and involve many parts of a person’s life, psychosocial and lifestyle factors play an important role in how well people with diabetes manage their disease (5).

Several psychosocial factors have been identified as barriers to good glycemic management, including fear of hypoglycemia, depression, lack of motivation, lack of support, and diabetes burnout (6). The American Diabetes Association reports that 18-45% of people with diabetes experience diabetes distress (5). Diabetes distress refers to the ongoing worries, fears, and challenges of managing a chronic disease, including treatment burden, risk of complications, potential loss of function, and access to care concerns (7). Studies have also shown that depression is 2-3 times more prevalent in people with diabetes compared to the population at large (8). In light of these findings, psychological care has become an important aspect to consider in the overall care for diabetes patients (9). Clinical psychologists in diabetes care teams can contribute by assessing and treating diabetes-related mental health problems, as well as help educate and advise other professions on how to support the psychological wellbeing of patients (9). It is also important for psychologists to be educated on diabetes to be able to understand and treat specific problem areas related to the disease (9).

In recent years, several studies have investigated the effect of different psychological interventions on glycemic management. Results have been mixed, with some studies finding effects on HbA1c and others not. In a recent review (10), no significant effect of psychological interventions was found on HbA1c in both children and adults with type 1 diabetes. Another review published in 2021 examined six randomized controlled trials on adolescents with type 1 diabetes (11). Three studies showed an improvement in glucose levels, while the other three found no significant changes (11). Further, a review from 2018 found psychological interventions that were specifically tailored to people with diabetes (both type 1 and type 2) were effective in improving both HbA1c and diabetes distress (3). A majority of the studies of psychological interventions for adults with type 1 diabetes were given in a group setting and were based on CBT (10). Randomized controlled studies evaluating the effect of individual CBT on HbA1c are however scarce, and the few studies that exist show mixed results. In a large study by Ismail et al. (12) the combination of individual motivational enhancement therapy (MET) and CBT was found to have a positive effect on HbA1c while several studies where CBT was evaluated for comorbid conditions in type 1 diabetes (13–16) found no effect on HbA1c (although on the comorbid condition).

To summarize, the scarcity of randomized controlled trials of the effect on HbA1c of individual CBT delivered by diabetes-educated psychologists suggest a need for more studies, to further increase our understanding of how and when psychological interventions based on CBT can help patients improve glycemic management.

The primary aim of this study is to assess whether treatment by a diabetes-educated psychologist can help patients with high glucose levels improve their HbA1c. A secondary aim is to investigate the effect of psychological treatment on diabetes distress and other psychosocial factors such as quality of life as well as treatment satisfaction. As mentioned above, diabetes distress is a common issue, and many people struggle with finding balance between diabetes and other areas of life. Should the results of this study show an improvement in blood glucose levels and/or psychosocial factors such as diabetes distress, it could be an incentive to start involving psychologists in the treatment of patients with diabetes on a broader scale. The results of this trial may also help guide future research in this area.

## 2 Materials and methods

### 2.1 Study design and locations

This is a non-blinded, randomized clinical trial with a parallel design. The study was conducted at six diabetes specialty clinics in Stockholm, Gothenburg, Uddevalla, Uppsala, Linköping, and Norrköping. Participants were recruited between January 2019 and December 2023. Results are anticipated to be presented in late 2025 or early 2026. Patients were initially contacted by written correspondence, followed by telephone contact. Patients were randomized 1:1 to either treatment with a diabetes-educated psychologist or conventional care. The study duration for each patient was 52 weeks. Psychological treatment consisted of a minimum of five visits with the psychologist in the first three months and a minimum of two follow-up visits in the following 9 months. A majority of visits were performed face-to-face at the diabetes out-patient clinics. Some visits were performed via video or telephone. A total of 10 diabetes-educated psychologists provided treatment in the study across all six clinics. Both individuals in treatment and control groups visited with a diabetes nurse at randomization, 3 months, 6 months, and 1 year. Extra visits with a diabetes nurse could be scheduled, if necessary, in the event of very high HbA1c. Both groups also met with a physician at the screening visit and at 1-year follow-up.

### 2.2 Eligibility criteria

All participants gave verbal and written informed consent before participating. The study was approved by the Swedish Ethical Review Authority.

The study included adults with type 1 diabetes and HbA1c >62 mmol/mol (7.8%). All inclusion and exclusion criteria are listed in **Table 1**.

**Table 1:** Inclusion/exclusion criteria.

Inclusion criteria
Informed consent obtained before trial-related activities Type 1 diabetes Age >18 years HbA1c >62 mmol/mol (7.8%)

Exclusion criteria
Type 2 diabetes Diabetes duration <1 year Long-term systemic glucocorticoid treatment during the last 3 months Treatment changes in the last 3 months (e.g., MDI vs insulin pump, started or stopped CGM or FGM therapy) Planned move or travel in the next 12 months that would interfere with study participation Current or planned pregnancy or breastfeeding during the next 12 months Other reasons determined by investigator (e.g., severe psychiatric conditions, alcohol/substance misuse etc.)

### 2.3 Randomization

Participants were randomized using a centralized web system that was handled by Statistiska Konsultgruppen Sweden. Every patient was assigned a unique and anonymous subject ID. The system was balanced for age, sex, and HbA1c.

### 2.4 Treatment

#### 2.4.1 Clinical visits

After inclusion and randomization, individuals in both treatment and control groups met with a diabetes nurse at 13, 26, and 52 weeks. A physician was also present for the inclusion visit and the 52-week follow-up where physical examinations were performed. At each follow-up visit HbA1c, blood pressure, and weight were measured. Insulin type and doses, type of glucose monitoring, and insulin delivery method were also checked, as well as the occurrence of adverse events. At randomization and at 26 and 52 weeks, diabetes distress was measured using the Diabetes Distress Scale (DDS) (17), which summarizes overall level of diabetes distress, as well as the level in specific areas. Quality of life was measured using the Audit of Diabetes Dependent Quality of Life (ADDQoL) (18), which measures present quality of life, the impact of diabetes on quality of life, and the average weighted impact of 19 different areas in life and how they are affected by diabetes. Hypoglycemia confidence was measured using the Hypoglycemia Confidence Scale (HCS) (19) which is a 9-item self-report scale which evaluates confidence in preventing and addressing hypoglycemic events, and treatment satisfaction was measured using the Diabetes Treatment Satisfaction Questionnaire status and change version (DTSQs and DTSQc) (20, 21), containing eight items scored on 7-point scales that are summed to a total treatment satisfaction score.

#### 2.4.2 Diabetes education for psychologists

All psychologists participating in the study were educated in cognitive behavioral therapy (CBT), which was the treatment of choice. Psychologists were also educated about type 1 diabetes before they could start treating patients within the study. The goal of the education was for the psychologists to have an overall understanding of the medical treatment of type 1 diabetes, as well as common psychosocial difficulties connected to the disease.

Education included seminars and self-studies about central aspects of diabetes care such as basic glucose physiology, hypoglycemia, insulin treatment (with injections or insulin pumps), risk factors, complications, glucose-monitoring and self-care recommendations. A psychologist with experience in treating patients with diabetes provided education on psychological aspects of diabetes treatment and common psychosocial barriers to glycemic management. All psychologists also participated in 20-25 clinical visits with a diabetes nurse or doctor. In addition, psychologists wore CGMs and insulin pumps (without insulin) themselves for 2-3 days in order to gain personal experience with some of the day-to-day tasks of living with diabetes, such as monitoring glucose levels and managing necessary medical equipment.

#### 2.4.3 Intervention by psychologist

Psychological treatment consisted of a minimum of five visits within the first three months and a minimum of two follow-up visits within nine months. However, more visits could be added if the psychologist and patient agreed on the need for more.

Psychologists were advised to start treatment with a clinical interview to gain understanding of participants’ individual circumstances and problem areas. A number of common psychosocial barriers to glycemic management were reviewed in the interview, see **Table 2**.

**Table 2:** Problem areas.

Problem area	Explanation
Depression	Current or previous depressive episodes
“No big deal”	Feeling that diabetes complications are not that serious
Inevitability	Feelings of hopelessness or that complications are inevitable regardless of one’s actions
Treatment skepticism	Dissatisfaction with, or lack of confidence in, one’s treatment plan
Poor social support	Lack of personal network to provide practical and emotional support
Unrealistic plans for action	Setting unrealistic expectations for oneself, e.g., always having perfect blood sugar levels, never eating anything unhealthy etc.
Environmental problems	Things in the home or work environment that make it difficult to take care of one’s diabetes
Denial of disease situation	Denial of the seriousness of the disease. Avoiding diabetes-related things or tasks, i.e., not looking at blood sugar levels, not attending hospital visits etc.
Lack of motivation	Struggling with motivating oneself to adopt necessary self-care behaviors
Problems regarding eating behaviors	E.g., eating large meals, eating even when one is not hungry etc.
Interpersonal or relational problems	Difficulties setting boundaries with others, or asking others for help when needed
Unrealistic fear of hypoglycemia	Avoiding low blood sugars, i.e., by not taking enough insulin. The fear is sometimes rooted in previous experiences.
Psychiatric conditions	Specific psychiatric conditions such as autism, ADHD, eating disorders, or personality disorders that may impact the ability to manage one’s diabetes

The interview also included screening questions for history of psychiatric problems. All participants completed the Patient Depression Questionnaire (PHQ-9) during the first visit with the psychologist (22), which is a tool for screening of depression. In the case of severe depression, the treating physician was consulted to determine where the patient should receive treatment.

The psychologist and participant formulated a treatment plan together, based on the problem areas identified in the interview, and reviewed during the course of treatment if necessary. CBT was the first choice of treatment, but other psychological treatment methods could be used if deemed appropriate by the psychologist. At specific diabetes-related questions the psychologist had the possibility to communicate with the diabetes nurse treating the patient. Visits were primarily performed as physical visits but could be performed digitally when needed.

### 2.5 Endpoints

The primary endpoint is the change in HbA1c from baseline to week 52. Secondary endpoints are changes in self-reported diabetes distress, quality of life, and treatment satisfaction. Primary, secondary, and exploratory endpoints are listed in **Table 3**.

**Table 3:** Endpoints.

Primary endpoint
Change in HbA1c from baseline to week 52

Secondary endpoints
Changes in DDS (Diabetes Distress Scale) score from baseline to week 52 Change in ADDQoL (Audit of Diabetes Dependent Quality of Life) score from baseline to week 52 DTSQc (Diabetes Treatment Satisfaction Questionnaire) score at week 52

Exploratory endpoints
Changes in DTSQs score from baseline to week 52 Change in HCQ (Hypoglycemia Confidence Questionnaire) score from baseline to week 52 Proportion of patients with HbA1c 58 mmol/mol (7.5%) or less at 52 weeks Proportion of patients with HbA1c 52 mmol/mol (6.9) or less at 52 weeks Proportion of patients lowering HbA1c 5 mmol/mol (0.46) or more from baseline to week 52 Change in total insulin dose from baseline to week 52 Change in weight from baseline to week 52 Change in blood pressure from baseline to week 52

### 2.6 Statistics

#### 2.6.1 Sample size calculation

The study is powered to detect an improvement in HbA1c of 4.4 mmol/mol (0.4 percentage units) from baseline to week 52. We assumed an SD of 8.7 mmol/mol (0.8%) for change in HbA1c for both treatment groups (23), showing that 64 individuals per group are needed to obtain a 80% power at an alpha level of 0.05. Accounting for a drop-out rate of 10%, 142 individuals were needed.

#### 2.6.2 Primary efficacy analysis

The primary efficacy analysis is the change in HbA1c from baseline to 52 weeks follow-up between the two groups using analysis of covariance (ANCOVA) with HbA1c at baseline as covariate in the ITT population, two-sided test of significance level 0.05.

#### 2.6.3 Secondary efficacy analysis

The secondary efficacy analysis is the change in diabetes distress scale (DDS) score from baseline to week 52 in the two treatment groups using ANCOVA with DDS score at baseline as the covariate. In case the assumption of normal distribution is not met, an effort to transform data to normal distribution will be made. If the assumption of normal distribution is still not met after transformation of data, logistic regression will be used with grouping variable as the dependent variable, change in DDS as main effect variable, and DDS score at baseline as the covariate.

Change in the quality-of-life score from baseline to week 52 between the two treatment groups will be analyzed using the same methodology as for the DDS score.

DTSQc score at week 52 between the two treatment groups will be analyzed using ANCOVA in case the assumption of normal distribution is met, or otherwise by using the Mann-Whitney U-test.

#### 2.6.4 Exploratory analyses

Exploratory endpoints will be analyzed in the same way as the primary and secondary endpoints, using ANCOVA for normally distributed variables, ANCOVA on transformed variables in case the assumption of normal distribution is not met, or logistic regression as described above. All exploratory endpoints will be adjusted for baseline values. Possible differences may exist depending on the distribution of variables.

An exploratory analysis is planned describing the psychological methods used by psychologists in the study and an analyses with respect to if results differed by psychologist, i.e. if an interaction exist between psychologist and treatment effect. We will also analyze if treatment effects relate to the number of consultations with psychologist.

## 3 Discussion

This paper describes the protocol of a randomized controlled trial investigating whether treatment with a diabetes-educated psychologist can help patients with type 1 diabetes improve glycemic management. Although HbA1c is the primary endpoint, an important objective is to investigate whether psychological treatment can help decrease diabetes distress, as many people with diabetes also struggle with this aspect of the disease.

Psychosocial factors such as depression, lack of motivation, and diabetes distress are recognized as important aspects that affect glucose management. However, psychologists are generally not involved in diabetes care teams to the same extent as other professionals such as nurses or dietitians. Previous studies have sought to investigate the effect of different psychological interventions on HbA1c. Interventions have varied from CBT-based treatments to motivational approaches and counselling. Some studies have found a significant improvement while other results have been mixed or shown no significant improvement (3, 10, 11).

Previous studies have assessed psychological interventions in both group and individual formats (3, 10, 11). Both treatment approaches have potential positive and negative aspects. Interventions given in a group setting can provide a sense of community and decrease feelings of loneliness or isolation. While individual treatment lacks the aspect of having input from other people in similar situations, it can more easily be focused on the specific problem areas of the patient. Additionally, some patients may not be comfortable attending group treatment for various reasons, including not wanting to discuss sensitive or emotional topics with others. Considering these aspects, an individual treatment approach was chosen for this study.

An important aspect of the present study was the diabetes education that psychologists received before treating patients. Education included basic information about diabetes, as well as specific psychosocial issues that many patients struggle with. It has been suggested that specific knowledge about the challenges of living with diabetes is important for psychologists to be able to identify problem areas and provide relevant treatment (9).

An aspect that can be seen as a limitation to this study is that psychological treatment is not strictly manual based, which could make replication difficult in future research, since treatment might not look the same for every participant. However, a number of guidelines were followed to secure quality and replicability of the intervention. For example, all patients were screened for depression before starting treatment. Psychologists also received a list of common psychosocial barriers for glycemic management that were reviewed with each patient at the beginning of treatment (**Table 2**) to identify focus areas. All psychologists were educated in CBT, which was the first choice of treatment before considering other methods. To make the intervention more representative of a real-life scenario, 10 different psychologists provided treatment. Within the framework of these guidelines, it was also important to allow for the treatment to be flexible and to be tailored to the needs of the individual patient.

In summary, this study seeks to investigate whether a diabetes-educated psychologist can help patients lower HbA1c levels and decrease diabetes distress. Should this study show beneficial results, it could provide justification for psychologists to become more involved in diabetes care going forward. On the other hand, improvements in diabetes distress or other psychosocial factors without significant effects on glucose levels will also be essential for patients in clinical practice. Either way, we hope this study will increase knowledge about how to help patients who struggle managing diabetes either physically or emotionally.

## Data Availability

The original contributions presented in the study are included in the article/supplementary material. Further inquiries can be directed to the corresponding author.
